# Iron redox pathway revealed in ferritin *via* electron transfer analysis

**DOI:** 10.1038/s41598-020-60640-z

**Published:** 2020-03-04

**Authors:** Peng Chen, Evelien De Meulenaere, Dimitri D. Deheyn, Prabhakar R. Bandaru

**Affiliations:** 1 Department of Mechanical Engineering, University of California, San Diego, La Jolla, CA 92093 USA; 2 Marine Biology Research Division, Scripps Institution of Oceanography, University of California, San Diego, La Jolla, CA 92037 USA

**Keywords:** Biophysical chemistry, Analytical biochemistry, Electrochemistry

## Abstract

Ferritin protein is involved in biological tissues in the storage and management of iron - an essential micro-nutrient in the majority of living systems. While there are extensive studies on iron-loaded ferritin, its functionality in iron delivery is not completely clear. Here, for the first time, differential pulse voltammetry (DPV) has been successfully adapted to address the challenge of resolving a cascade of fast and co-occurring redox steps in enzymatic systems such as ferritin. Using DPV, comparative analysis of ferritins from two evolutionary-distant organisms has allowed us to propose a stepwise resolution for the complex mix of concurrent redox steps that is inherent to ferritins and to fine-tune the structure-function relationship of each redox step. Indeed, the cyclic conversion between Fe^3+^ and Fe^2+^ as well as the different oxidative steps of the various ferroxidase centers already known in ferritins were successfully discriminated, bringing new evidence that both the 3-fold and 4-fold channels can be functional in ferritin.

## Introduction

Ferritin is an ubiquitous protein^1,2^ involved in the storage and management of iron - an essential micro-nutrient for almost all living systems. The protein occurs in abundance in the cytosol and mitochondria, where it helps maintaining the performance of critical biochemical reactions^3–6^ and balance oxidative stress processes^7^. Ferritin is therefore mainly internal in most systems, but can sometimes be a secreted enzyme^7^, and can thus be used to manage balance of iron both intra- and extracellularly, which can protect from deleterious excess of iron uptake, but also from viral and bacterial infections. Ferritin is known to take up iron (as Fe^2+^) and store it in stable unreactive Fe^3+^-oxide/hydroxide form (primarily ferrihydrite). It is believed to deliver Fe back in a functional Fe^2+^ form, where and when needed for biological processes^8^. In spite of many biochemical studies on performance of loaded ferritin, some parts of the molecular mechanisms of iron uptake, *e.g*., associated with oxidation steps, and most of the iron release and delivery, *e.g*., associated with reduction steps), remain largely unclear and speculative^8–11^.

**Figure 1:**
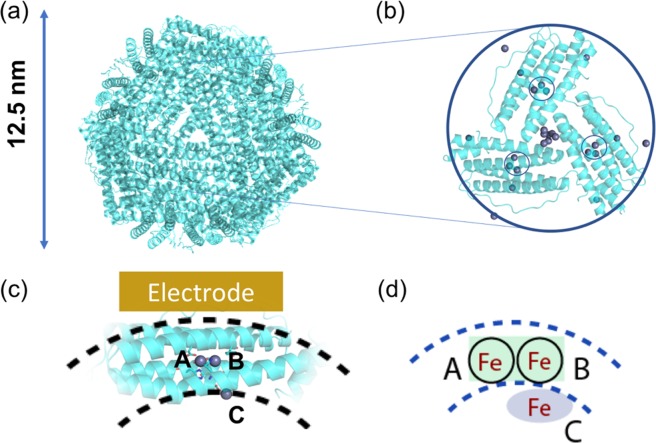
Ferritin structure and related iron binding sites, **(a)** The crystal structure of a ferritin cage, with the 24-subunit ensemble spanning ~12.5 nm. Each subunit carries a ferroxidase center buried inside a four-helix bundle. **(b)** Three ferritin sub-units indicating the four-helix bundles delimiting a 3-fold channel and displaying metal ions (purple) at the location of the ion channel and the ferroxidase site (indicated with circles). **(c)** The Fe^2+^/Fe^3+^ binding sites: A and B constitute the ferroxidase center, and site C is a ferrihydrite nucleation/gateway site. The dashed lines represent the cross section of the 24-meric cage (shell). **(d)** A schematic representation of the A, B, and C sites, within a ferritin shell. All crystal structure representations are made in PyMol^38^ based on 2CIH.pdb^39^.

Structurally, ferritin is a biomacropolymeric enzyme constituted of 24 subunits (Fig. 1a,b) forming a hollow structure – often referred to as a cage that can be loaded^12^ with Fe. The cage has an octahedral (432) symmetry where the 4-fold and 3-fold axes of symmetry are the place where 4 and 3 subunits meet together to respectively delineate six four-fold channels and eight three-fold channels. These channels have a small diameter (ranging from ~0.2 nm to ~0.5 nm) that varies along the 3 - 4 nm length of the channels. To date both the 3-fold and 4-fold channels have been considered for the Fe^2+^ ion entry into the core of the cage, but proof of natural activity^8,11,13^ has been delivered only for the 3-fold channels. There are three types of ferritin subunits: Light (L), a Middle (M) and a Heavy (H) subunit, in increasing order of molecular weight. Ferritin cages are usually constituted of a mix of Light and Heavy chain ferritins. Other ferritins only consist of a single type similar to the heavy chain. In the center of all except the L-type ferritin subunit, a ferroxidase site (capable of oxidation and possibly reduction) was identified through multiple mutation studies^8^, containing two prominent Fe binding and oxidation sites, named site A and B (after their affinity for Fe)^13^: Fig. 1c,d. A third metal binding site is observed in several crystal structures, referred to as the C site. This C site is not usually considered associated with ferroxidase activity but would play a role as a gateway in the passage of the iron as a transient form to the ferrihydrite stage for final storage^8^.

The amino acids interacting with the metal ions in these three key sites delineate the ferroxidase site in which the oxidation of Fe^2+^ to Fe^3+^ takes place when taking up iron, and potentially also the reductive step of Fe^3+^ to Fe^2+^ when releasing, although some ambiguity exists on the exact mechanisms related to the redox dynamics^14,15^, especially when considered under the influence of external reducing agents^16^.

Aside from bacterioferritins, most studies on Fe ion dynamics have been on horse (equine) ferritin because of large availability, as well as on human ferritins because of the incentive to understand the critical role of ferritin in human diseases^17^. Consequently, crystal structures of less than 20 different eukaryotic ferritins are available, and both structures and kinetics studies of homopolymers across species have always reported similar properties, thus rendering the establishment of natural structure-function relationship relative to performance rather challenging, but also irrelevant. After all, the ferritin system machinery, as found in most eukaryotes appeared to have evolved to the ultimate perfection and most mutations studied are deleterious.

Recently, a ferritin (*Chaetopterus* ferritin, ChF) was found associated with the production of visible light (bioluminescence) in the secreted mucus^18,19^ of the marine tube worm *Chaetopterus sp*^18^. Interestingly, this was the first natural eukaryotic ferritin found to significantly outperform human heavy chain ferritin (HuHF) - a conventionally accepted representative of a vertebrate ferritin, for ferroxidation velocity, being up to *eight* times faster^19^. This discovery however provided the first natural eukaryotic “out of the ordinary” ferritin that could be used as a tool to understand the mechanistic pathways in ferritins, which could be critical for some medical as well as biotechnological applications^20–23^.

From a biochemical point of view, the Fe^2+^ transportation mechanisms *into* and *out of* the ferritin core^2^, and the related electron movement associated with reduction steps, are not yet entirely understood^13,24^. A better understanding of charge transfer processes is clearly needed to pave the way for a more complete assessment of ferritin functionality and establish structure-function relationships in relation to electron transfer at the molecular level. Mossbauer spectroscopy^25^ as well as EPR-spectroscopy^26^ have been previously applied to study ferritin-related mechanisms. While the iron oxidation states inside the ferroxidase center were revealed, the path of electron transfer remained unclear from these analyses. Electrochemical methods such as cyclic voltammetry have also been applied to study molecular chemistry of ferritins, yet due to the difficulty of observing charge transfer between electrode and ferritins, such experiments had to be performed using redox mediators, thus making the identification of electron paths challenging^27^.

To probe the redox mechanism of the high-performing ChF ferritin, we decided to use voltammetry methods. Since the kinetic processes in ferritin are quite rapid (within 50 ms)^28^, conventional voltammetry methods such as cyclic voltammetry with a fast scan rate (>50 V/s) were deemed insufficient. The approach of differential pulse voltammetry (DPV) was applied instead to measure and integrate coupled processes related to ferritin species related transport to the electrode^16^. While DPV is developed for the measurement of small concentrations, we here take advantage of the stepwise pulsed voltage increases to enhance the sensitivity and specificity of the response and help address the issues related to deformation and adsorption of ferritin protein to electrodes surface. Considering the relative structural complexity of the ferritin protein and that the active ferroxidase sites are buried relatively deep within the ferritin, one can assume ferritin related voltammetry^29^ to be integrating multiple scales and types of events, *e.g*., related to charged species transport to the electrode, relevant enzyme kinetics, as well as interfacial electron exchange^30^ each of which rate limiting in its own time and spatial scale^31^.

In this work, we combine comparative analysis of HuHF and ChF crystal structures with electrochemical analysis aiming to (1) better understand the flow of electrons in both ferritins in association with the DPV redox kinetics of iron, as well as to (2) determine which electrochemical step and/or pathway of the ferritins are different enough to sustain the measured difference in ferroxidase activity between HuHF and ChF. We decided to use the homomultimeric form of HuHF rather than work with a mix containing light chain ferritin, in order to make sure measurements were made on a simplified system with no protein-protein interactions (between heavy and light chains) that could interfere with our data interpretation.

## Results and Discussion

Voltammetry of the ferritin complex measures and integrates coupled processes related to charged species transport to the electrode, relevant enzyme kinetics, as well as interfacial electron exchange – each of which may be rate limiting^30^. The redox kinetics of ferritin were previously recorded as subject to a variety of factors, including pH, surrounding buffer content, and long-range electron transfer through the protein matrix^16^. Our approach primarily consists of the parameterization of redox behavior in terms of the relative number of the Fe ion species (Fe^2+^ or Fe^3+^) with respect to the protein. In the work reported here, recombinant ferritin with around 10 Fe ions per cage was used to probe for charge transfer between ferroxidase center and electrode. The core Fe related processes were not considered in our investigations. As indicated in the Methods section, voltammetry measurements from native ferritin (~10 Fe/cage average load) and slightly loaded ferritin (31–32 Fe/cage average load) showed identical peaks (also see Section 4 in *Supplementary Information*).

**Figure 2:**
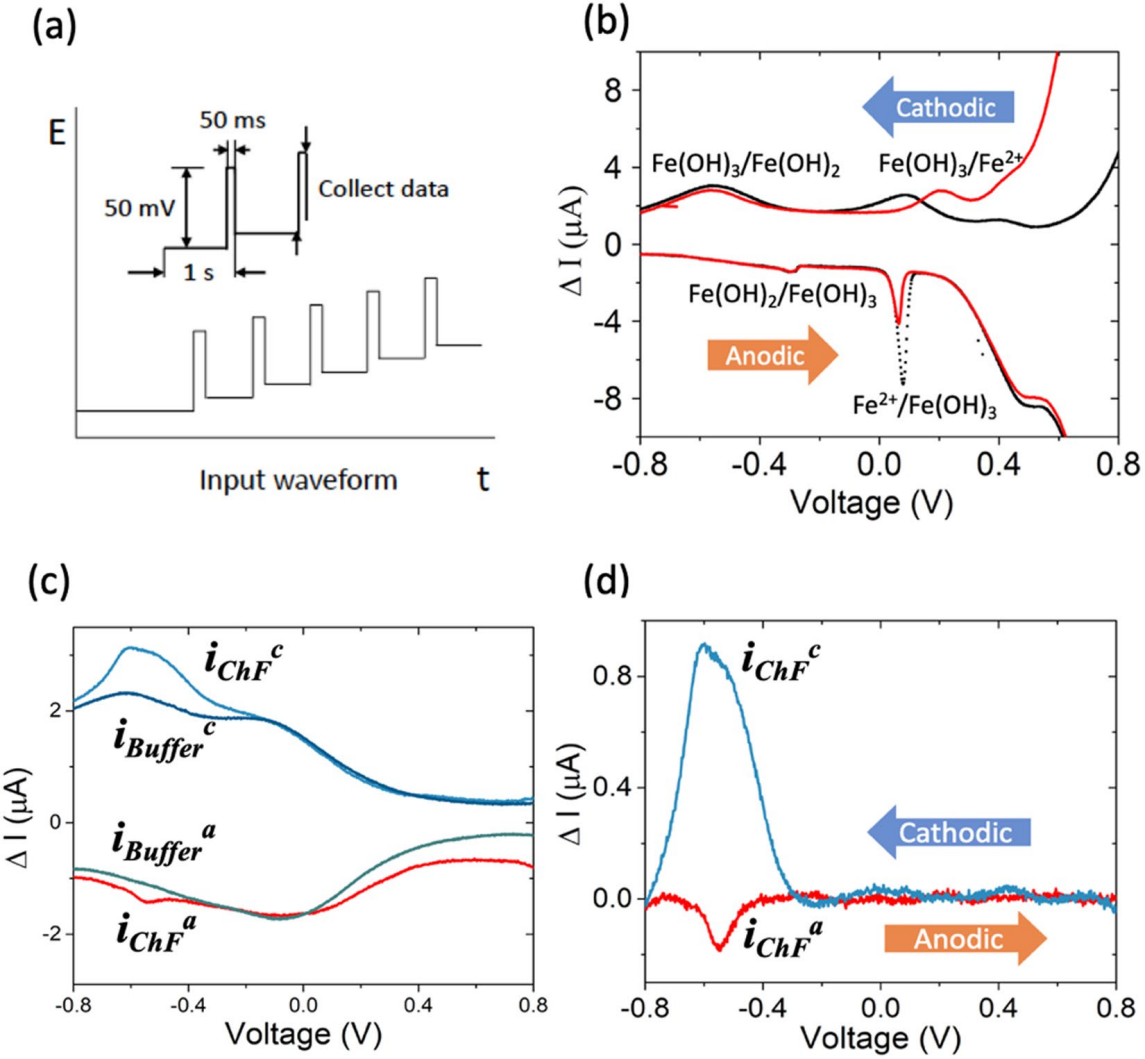
Differential pulse voltammetry (DPV) was used for the interrogation of the redox characteristics of the ferritin and incorporates **(a)** the application of a voltage pulse (of typical magnitude *V*_*p*_ of ~50 mV, and applied for ~100 ms, in our experiments), superposed on a steadily increasing base voltage (*V*_*base*_). The difference in the electrical current *before* and *after* the voltage pulse is a measure of the Faradic response of the ferritin and is manifested through an electrical current peak corresponding to the relevant redox (reduction/oxidation) reactions. **(b)** DPV scans of FeCl_2_ in aqueous solution. **Black**, pH ~7, and **Red**, pH ~14. The redox peaks, observed in the cathodic scan, correspond broadly to the Fe^3+^ → Fe^2+^ reduction processes, and assigned to the Fe (OH)_3_/Fe (OH)_2_. In the reverse voltage scan, anodic redox peaks arise from the Fe^2+^ → Fe^3+^ oxidation processes and are assigned to the Fe^2+^/Fe (OH)_3_ **(c)** A comparative DPV with cathodic (*c*) and anodic (*a*) scans of *ChF* (in a phosphate buffer and NaCl) – outer curves, and the *Buffer* (without ChF) – inner curves. The subtraction of the *Buffer* scans from the observed signals from the combined ChF + *Buffer* yield a clearer delineation of the peaks belonging to the ChF alone. **(d)** Same raw data as from panel (c) but after *Buffer* background signal subtraction. This last format is how voltammograms will be presented in the remaining of this work.

Differential pulse voltammetry (DPV), was selected to probe the electrochemical characteristics of the ferritins with increased species-specific sensitivity^32^. Description and interpretation of typical recordings of the voltammograms are detailed in Fig. 2 and in the Methods section.

Control experiments were performed to identify potential contributions from apoferritin (without Fe inside the core), free Fe^2+^ ions as well as ions bound to hydroxides, from those related to the ferritin bound Fe^2+^/Fe^3+^ ions (see Figure S1 in *Supplementary Information*). Figure 2(b) indicates the redox processes obtained, in the cathodic and anodic scans, by adding FeCl_2_ yielding Fe (OH)_3_/Fe (OH)_2_ pair in aqueous background solutions. The labeled redox peaks observed in the low voltage range (centered at −0.54 V) are ascribed to the redox pair Fe (OH)_3_/Fe (OH)_2_, and the redox peaks centered at 0.2 V are ascribed to the Fe^2+^/Fe (OH)_3_. Free Fe^2+^ ions are unstable at pH = 7, where they undergo auto-oxidation and precipitate into ferric hydroxide. A ChF ferritin DPV signal - outer lines in Fig. 2(c) was clearly measured and distinct from the background baseline, which is the phosphate buffer by itself - inner lines in Fig. 2(c).

The ChF ferritin DPV signal appeared with more details (Fig. 2d) when the background (buffer) was subtracted from the signal curves. The redox potential measured is quite close to that of Fe (OH)_3_/Fe (OH)_2_ and such a redox pair only exists in the ferritin with iron ions (See Figure S1 in *Supplementary Information* for comparison with apoferritin). Supporting our findings, another mechanistic study^25^ yielded similar redox potentials, confirming that the signal detected in our ferritin samples is indeed from iron going through chemical transformation inside the ferritins. Consequently, our results report with high fidelity Fe ion redox dynamics *internal only* to the ferritin.

**Figure 3:**
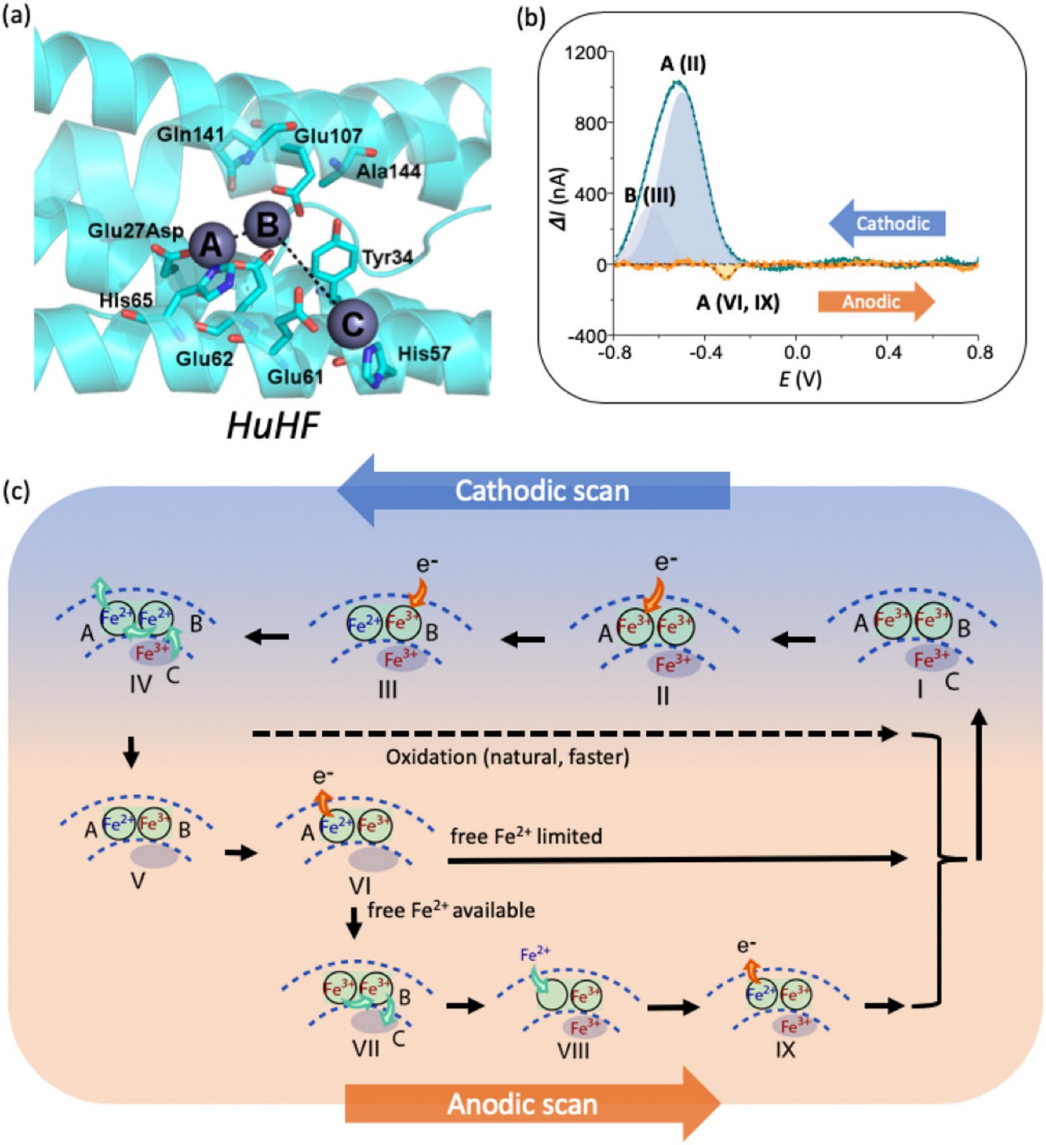
Redox kinetics in HuHF probed through differential pulse voltammetry and proposed redox mechanisms. (**a**) The HuHF ferroxidase center within a single subunit, indicating key residues and A, B and C sites. The respective distances between the metal ions are: A-B 3.5 Å, B-C 9.2 Å. PyMol^38^ was used to visualize HuHF with Zn in all 3 sites from 2CIH.pdb, despite the Glu27Asp mutation, **(b)** DPV derived peaks for HuHF, cathodic scan read first. The cathodic peak may be deconvolved to yield two contributions, related to the A and B sites of the ferritin, respectively, and the anodic peak is paired to the A peak. Roman numerals refer to relevant processes depicted in **(c) -** proposed mechanism for charge transfer processes in HuHF, as derived from the measured voltammograms. Orange arrows indicate the direction of electron flow, while green arrows indicate iron ion flow. The cathodic processes (reduction, *top*) at the two Fe^3+^ (in red) ions associated with the (II) A and (III) B sites yield two peaks corresponding to the creation of two Fe^2+^ ions (in blue) at the end of the cathodic scan. (IV) One of the formed Fe^2+^ is transported away from the ferritin and is (V) replaced by the Fe^3+^ from the C site or from the core (no specific proof of this was found in DPV). In the voltage-driven anodic process (*bottom*), (VI) the residual Fe^2+^ is oxidized to Fe^3+^ at site A only (when little Fe^2+^ is available) and constitutes the one peak observed in the anodic scan. (VII) The rearrangement of Fe^3+^ supplies the iron to the C site or to the core. Depending on the abundance of external free Fe^2+^ (limiting vs not limiting), the ferroxidase site can *either* remain vacant with limited free Fe^2+^ *or* take up iron and oxidize it when free Fe^2+^ is available abundantly^8^, as shown in (VIII) and (IX). Three possible pathways have been indicated for the oxidation. The top path (in dashed lines) represents the fast, natural oxidation taking place inside the ferritin in the time frame between the cathodic and anodic scans. The two others are the voltage-driven reactions, associated with the voltammogram.

We first discuss the DPV involving dissolved HuHF (Fig. 3), and then subsequently compare with the kinetically faster ChF. When following the voltammogram of HuHF (Fig. 3b), which started with a cathodic scan, followed by an anodic scan, the reduction and oxidation signals related to the iron inside the ferritin appeared at a pronounced current change, at the relevant applied voltage. The cathodic peak was deconvoluted into two subsidiary peaks designated A and B (Fig. 3b); peak B being at slightly more negative voltage than peak A. The basis for the deconvolution was the asymmetrical shape of the cathodic peak in both the HuHF and ChF DPV. We used appropriate peak fitting procedures, *e.g*., using second order derivatives, and OriginLab multi-peak fitting tools to yield two sharp peaks related to the A and B peaks in the cathodic scan, while such peak delineation was absent in the anodic scan.

**Figure 4:**
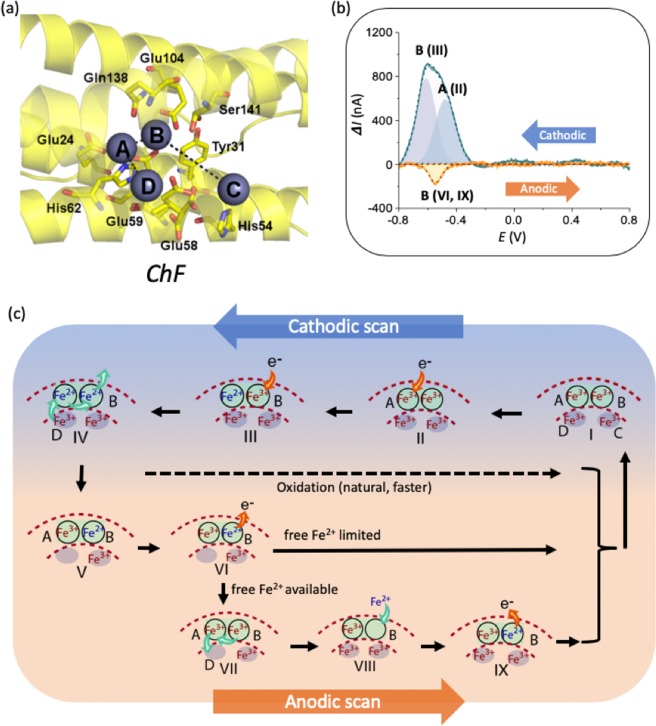
Redox kinetics in *Chaetopterus* ferritin (ChF) probed through differential pulse voltammetry and proposed iron redox mechanisms. (**a**) The ferroxidase center structure in ChF, showing all key residues including Ser141. The respective distances between the metal ions are: D-A 6.3 Å, A-B 3.4 Å, B-C 9.3 Å. ChF structure shown from 5WPN.pdb and visualized in PyMol^38^. An additional metal binding site near the ferroxidase center is found in 5WPN and labeled as site D. **(b)** DPV derived peaks for ChF, in the cathodic and anodic scans. The cathodic peak is deconvolved to yield contributions related to the A and B sites of the ferritin. The anodic oxidation peak is associated with the B peak only. **(c)** The hypothesized mechanism for the charge transfer processes related to the Fe redox kinetics in ChF based on the DPV scan. The cathodic processes (*top*) at the two Fe^3+^ ions are associated successively with the (II) A and (III) B sites and yield two peaks corresponding to the Fe^2+^ and Fe^3+^ rearrangement at the end of the cathodic scan (IV), involving the D site. One of the formed Fe^2+^ ions is then transported away from the ferritin and (V) site A is occupied by Fe^3+^ from inside the ferritin. In the anodic oxidation process, (VI) the residual Fe^2+^ is oxidized to the Fe^3+^ in site B and constitutes the one peak observed in the anodic scans. (VII) The oxidized iron supplies the Fe^3+^ to the inner iron site. (VIII) and (IX) are follow-up steps when free Fe^2+^ is available.

We propose peak A and peak B to be associated with Fe bound in sites A and B of the ferroxidase center^8,33^, respectively; *cf*. Figures 1(c,d) and 3(a) - also see Figure S2 in the *Supplementary Information*. While the two iron atoms in the ferroxidase center have similar chemical environment, the B site is coordinated through acidic groups (Glutamates) only, in contrast with the A site that is coordinated through acidic as well as an alkali groups (Histidine 65)^13,19,24^. This results in making the site B site slightly more electronegative than site A and is invoked to explain the different peak positions observed in the voltammograms: Figs. 3(b) and 4(b). After reduction in the initial cathodic scan, Fe^2+^ is re-oxidized by the ferritin and potentially even auto-oxidized in the buffer, leaving only a small amount for the oxidation during the anodic scan, resulting in a smaller amplitude of the anodic peak. The anodic peak cannot be deconvoluted at this stage and was labeled “peak A”, since the only logical and coherent option in relation to the peak A observed in the preceding cathodic scan, and in line with expectations of closely spaced redox pairs^34^.

The HuHF voltammogram showed a relatively large peak separation of ~200 mV between the anodic and cathodic peaks (designated A), indicating quasi-reversible behavior^35,36^, and attributed to competing spontaneous Fe^2+^ oxidation by the ferritin. These observations are in agreement with an EC mechanism, referring to an electrochemical (E) process followed by a chemically coupled (C) electron transfer process^37^. In our case, this is most likely driven mainly through the fast re-oxidation of Fe^2+^ by the ferritin occurring spontaneously before the scan reaches the anodic peak voltage (as opposed to be representative of the auto-oxidation process in the buffer that is much slower and providing much less significant signal in the voltammograms). Ferritin can oxidize Fe^2+^ in a millisecond time scale, much faster than the time elapsed between oxidation and reduction peaks in the DPV scans. The location of the anodic peak seems to indicate that oxidation happens in the A site only, at least when availability of Fe^2+^ ions is limited. Our data provide the first direct demonstration of what has been only speculated on thus far in the literature.

The proposed mechanisms for the observation of the cathodic and anodic features are indicated in Fig. 3. Conventionally, it is believed that the 3-fold channel is the path of Fe^2+^ entry, after which an electrostatic gradient guides Fe^2+^ ions from the channel to the ferroxidase site^8^. In this regime, iron ions are delivered into site A first, then passed on to site B, and finally stored in the core. The involvement of the C site is unclear although it is usually referred to as the ferrihydrite nucleation site or gateway site, shuttling Fe ions between the catalytic center and the core, in spite of indication of ferroxidase activity^8^.

In the DPV performed on ChF, the deconvolution of the cathodic peak shows the peak associated with the A site to be smaller than the one associated with the B site: Fig. 4(b), opposite to the HuHF situation (compare relative height of subsidiary peaks A and B in Figs. 3(b) and 4(b). Moreover, the single oxidation peak observed in the anodic scan, was identified as a peak B from the expected proximal and oppositely located cathodic and anodic peaks positions. Thus, the anodic ChF peak can only be explained if associated with the cathodic B peak. The microenvironment in the respective active sites is identical between HuHF and ChF, except for residue Ser141 in ChF vs Ala144 in HuHF (also see Figures S2 and S3 in the *Supplementary Information*). The residue is proximate to the B site - see Fig. 4(a), and Ser141 may be providing additional stabilization to Fe in site B. In the 5WPN crystal structure of ChF, a water molecule is found between Ser141 and the Fe B site. Ser141 exists in a dual conformation with the hydroxide at a 3.0 Å or 3.3 Å distance from the water molecule, which is positioned at 2.0 Å from the Zn ion in the structure (Figure S3). This water molecule most likely contributes to the increased stability, which could explain the increased cathode current peak amplitude in the ChF voltammogram compared to that seen in HuHF. The oxidation peak being related to the Β peak makes a different oxidation pathway than indicated for HuHF seem more plausible, as is shown in the corresponding panels (VIII) and (IX) of Fig. 4(c) and described below.

Like in the HuHF schematic, three possible routes are shown (Fig. 4c) for the anodic step, *i.e*., the top (dashed line) represents the fast, natural oxidation taking place inside the ferritin in the time frame between the cathodic and anodic scans. The other two are associated with voltage-driven oxidation/reactions. When free iron is limited, oxidation is being considered to take place in the B site only. When Fe^2+^ is available more abundantly, a sequential process (VII- IX) occurs, here adapted for initial oxidation in the B site^38^.

Integral to the alternative pathway is another metal binding site - arbitrarily labeled “D” in Fig. 4a, proximate to the A site, and found in the ChF crystal structure^19^. Comparison with over 30 other available crystal structures of eukaryotic (heavy chain) ferritins shows that this is a unique configuration (also see section S3 of the *Supplementary Information*). As is often done in enzyme crystallography, Zn^2+^ was used as an inert substrate alternative with comparable size and charge as Fe^2+^ in the generation of the relevant 5WPN crystal structure^19,39^. While this has proven to be a conventional approach for optimization of structural analyses, interpretation relative to the electrochemical function of structural features can be more challenging. For example, despite similar structural positioning of the A-B sites in the ferritins, the voltammogram for ChF (Fig. 4b) shows oxidation in the B site first, which can only could be rationalized by a pathway inside the enzyme involving the entry of Fe^2+^ ions into the ferroxidase site through the B site first, *instead* of the A site and oxidizing the Fe^2+^ ions in the B site such as for HuHF: Fig. 4(b). Ideally, in order for this mechanism to work while keeping the enzyme configuration in mind, the Fe ions have to enter and exit the ferroxidase center on the opposite side in comparison to HuHF: Fig. 3(c). Interestingly, it has been previously postulated^8^ that site B may be accessible through the four-fold channel which has been proven a possibility through mutation studies^40,41^. The activation of the four-fold channel in bullfrog ferritin enhanced the ferroxidase activity five-fold. A comparable enhanced performance has been previously observed naturally in the wild type ChF^19^. Indeed, a detailed comparison of the crystal structure of HuHF (Figure S4 in *Supplementary Information*) reveals such a Glutamate and hydroxide rich path between the four-fold channel and the ferroxidase center, only in the faster ChF. As for metal ions exiting the ferroxidase site on the opposite side, the D site in the ChF crystal structure could be assigned the gateway site in this new pathway. Clearly this difference in electro-chemical mechanisms across sites between ChF and HuHF could only be detected using DPV and not structural analyses only.

While previous work in our group^19^ has shown that ChF takes up iron considerably faster than HuHF, the present study shows that the related mechanism might be more drastically different than initially anticipated. The faster kinetics are not just explained by a few residues in the commonly accepted pathway but by a *complete shift* in the pathway to a faster entry for Fe^2+^ ions. Our study points out that the oxidation takes place in different sites (A *vs*. B) in the ferroxidase site in the ferritins, which can only be explained by entry of the Fe^2+^ ions from the opposite side (towards B site, instead of A), which is feasible through invoking the four-fold channel in ChF. After oxidation, the Fe^3+^ ions would exit the active site from the A site through the newly assigned D site and find their way to the core. Through voltammetric analyses, we were able to provide proof of the reducing capabilities of both sites in the ferroxidase center in both ferritins, reveal their differences, and shed light on the question as to where in the ferroxidase center the oxidation takes place and/or in which order.

The reported work yields considerable insight related to the molecular mechanisms of ferroxidase activity in ferritins. The benefit of integrating analyses for tailored biochemical performances across different animal species was demonstrated, where ferritin operates under different environmental constraints, through DPV - which for the first time was used outside its conventional application, here to resolve redox mechanisms. Consequently, it was possible to provide step-by-step identification of the reduction and oxidation pathways of iron in ferritin, and that the sequence of oxidation steps might not necessarily be preserved across ferritins. It was concluded for example that both 3-fold as well as 4-fold channels can have active roles in iron transfer. It was also posited that small, albeit targeted, differences in residues between ferritins can lead to significant changes in electrochemical and biochemical performances. Our work thus opens the door to more applied research where directed changes of specific residues could be used to increase performance of human ferritin when competition for iron resource and/or access to limited iron are conditions deemed to have critical health impact.

## Methods

### Sample preparation

All ferritin samples were obtained by expression in *Escherichia coli* and purification of the lysate using liquid chromatography following conventional protocols:

#### Cloning and expression

The gene for ChF was obtained through amplification of *Chaetopterus* cDNA^18^ and cloned into the multiple cloning site of a pET24b vector using *NdeI* and *BamHI* restriction sites (using forward primer: CACAAGATCATATGGCCCAGACTCAGCCG and reverse primer: GTCGTGGATCCTTAGCTGCTCAGGCTCTCCTTGT). The gene for HuHF wild type was obtained through site directed mutagenesis of a codon optimized HuHF ΔC* mutant (in which all cysteines are replaced by alanine) in a pJexpress414 vector from DNA 2.0 (Menlo Park, CA) as previously described^19^. After transformation, BL21 star (Invitrogen) cultures were grown at 37 °C to an OD_600 nm_ of 0.7–0.8, induced with isopropyl β-D-1-thiogalactopyranoside (IPTG) and the proteins were expressed for 8–10 h after induction. The cells were harvested by centrifugation at 4,000 rcf for 25 mins at 4 °C. The cell pellet was resuspended in a 25 mM TRIS (tris(hydroxymethyl)aminomethane) buffer at pH 8.0 with 160 mM NaCl and lysed using lysozyme (Sigma) and Benzonase nuclease (EMD Millipore) at 37 °C for 30 min each, followed by sonication on ice for 3 min with 0.5 s intervals.

#### Purification

The resulting lysate was incubated in a warm water bath at 75 °C for 20 mins, followed by centrifugation at 4,000 rcf for 15 min to remove denatured protein. The remaining lysate was diluted in 25 mM TRIS buffer (pH 8.0) without NaCl and loaded onto a HisTrap Q HP column and eluted using a linearly increasing NaCl gradient. Of each collected fraction, 10 μL was tested for ferritin activity by adding 30 μL 2 mM FeCl_2_ and 120 μL MES buffer (20 mM MES, 200 mM NaCl, pH 6.85). After 20 minutes of incubation at room temperature, 20 μL FerroZine (1 mM) was added and all active samples (colorless or as determined by absorbance measurement at 562 nm in SpectraMax (Molecular Devices)) were pooled, concentrated in spin concentrators (Sartorius, molecular weight cut-off, MWCO: 50,000 Da) and further purified through gel filtration on a Superdex 200 column in a 20 mM MOPS buffer with 150 mM NaCl at pH 6.5. All gel filtration fractions were also tested, and active fractions were selected and combined. All samples were further concentrated, and buffer exchanged to a Na_2_HPO_4_ buffer (50 mM Na_2_HPO_4_, 200 mM NaCl, pH 7.6) using spin concentrators with a MWCO of 50 kDa (Sartorius).

### Iron determination

The average iron content of the ferritin cages was determined using Inductively Coupled Plasma Mass Spectrometry (ICP-MS). The protein samples used for DPV measurements were first diluted to around 1 mg/mL for protein determination by Bradford method. The ~1 mg/mL samples were further diluted 100 times in 2% HNO_3_ for the ICP-MS measurements. The Fe content was measured on an iCAPQc Single Quadrupole ICP-MS instrument (Thermo Fisher Scientific) with a 0.05 s dwell time and the analysis validated against the Fe content in a calibration series ranging from 1 to 1,000 ppb Fe standard (Table S1 in *Supplementary Information*), as routinely done in the elemental isotopic research facility (SIO). From these ICP-MS and Bradford measurements combined we obtained the total iron content per ferritin cage being between 8.5 and 10.5 Fe atoms for the WT samples before loading with FeCl_2_ (see Table S2 in *Supplementary Information*). Ferritin with slightly higher Fe content was prepared by adding 100 μL of 10 mM FeCl_2_ to 1 mL ferritin solution of 20 mg/mL. Voltammetry was performed during loading as well as 48 hours after loading. The average load per ferritin cage at that point is estimated at ~31–32 Fe per cage.

Voltammetry measurements from native ferritin (~10 Fe/cage average load) and slightly loaded ferritin (31–32 Fe/cage average load) showed identical peaks, as demonstrated in *Supplementary Information* (Section 4). For simplicity, the main text only shows results for the native ferritin.

### Differential Pulse Voltammetry (DPV) and interpretation

DPV was employed to probe the redox processes in solution and for obtaining high species-specific sensitivity^32^. Here, we probe the electrochemical characteristics of the ferritins (25 mg/mL with ~9–10 Fe per cage), where the redox behavior is parameterized in terms of the relative abundance of the iron ion species (Fe^2+^ or Fe^3+^). The aim was to understand the performance of *native* ferritin (with ~9–10 Fe per cage) and focus on the redox processes associated with the Fe only at the ferroxidase centers. The core ion related processes were not investigated, avoiding the complication of a large ferrihydrite core. Moreover, the Fe content (~9–10 Fe/cage) gave results similar to what was observed with higher loading (with ~31–32 Fe per cage) – as indicated in Section 4 of the *Supplementary Information*. As the DPV scans look similar to what was observed in the native ferritin case, *i.e*., Figures S5 in the *Supplementary Information*, we deem our native ferritin-based experiments to be representative of the redox processes and mechanisms indicated here.

The background electrolyte was a phosphate buffer (pH ~7.6) and 0.2 M NaCl to keep a moderate ionic strength^37^ and to guarantee stability and activity of the enzyme^19^. The ferritins (as also discussed in the previous section) were interrogated through suspension in the electrolyte, in the absence of background chelators, with a glassy carbon electrode (GCE), which served as the working electrode. A Pt wire and a saturated calomel electrode (*SCE*) functioned as the counter electrode and the reference electrode, respectively. An electrical current peak, in response to a voltage pulse, is a measure of the Faradic response of the ferritin, and probes both iron reduction (/oxidation) in cathodic (/anodic), with decreasing (/increasing) applied voltages (Fig. 2a).

This voltammetric measurement incorporates the application of a voltage pulse (of typical magnitude *V*_*p*_ of ~50 mV, and applied for ~100 ms, in our experiments), superposed on a steadily increasing base voltage (*V*_*base*_). Step size was selected as 2 mV and sample period was 1 s.

The difference in the electrical current *before* and *after* the voltage pulse is recorded and is a measure of the Faradic response. The *V*_*base*_ is increased from below the redox potential (*E*_*redox*_) to a larger value; at lower voltages, inadequate Faradic processes result in a low sampled current, while at voltages larger than *E*_*redox*_, the current is diffusion limited and low. In the intermediate regime, an electrical current peak is manifested corresponding to the relevant redox reaction. Moreover, taking the difference of the electrical currents at relatively close values of the voltage in a time interval, ensures adequate subtraction of the background contributions. See Fig. 2 and related text, for details related to the voltammetric procedures.

Consideration of the attributes such as the (i) ratio of the peak **A**nodic current (*i*_*p*_^*A*^) to the peak **C**athodic current (*i*_*p*_^*C*^), (ii) the respective half-peak widths (*W*_*1/2*_^*A*^ and *W*_*1/2*_^*C*^), (iii) the separation: *ΔE*_*p*_ (= |*E*_*p*_^*A*^ – *E*_*p*_^*C*^|) between the anodic peak voltage (*E*_*p*_^*A*^) and the cathodic peak voltage (*E*_*p*_^*C*^), have been used to indicate the electrochemical kinetics and related mechanisms^32,34^. An *i*_*p*_^*A*^/*i*_*p*_^*C*^ ratio closer to unity imply a reversible, single-electron transfer, electrochemical (E) process, while a ratio less (/more) than unity connotes an underlying EC (/CE) mechanism, where the following (/preceding) C indicates a chemically coupled electron transfer process.^29,42,43^, for instance, as in leading to iron storage compound formation. Correspondingly, a *W*_*1/2*_^*A*^/*W*_*1/2*_^*C*^ ratio equal to unity implies E processes, while a ratio more (/less) than unity posits an EC (/CE) mechanism^32,44^. Such analyses probe new frontiers in ferritin protein function, yielding insights into related electron transfer mechanism.

## Supplementary information

Supplementary information.
