# Supplementary material for: Iron redox pathway revealed in ferritin via electron transfer analysis
Source: Sci Rep. 2020 Mar 4;10:4033. doi: 10.1038/s41598-020-60640-z (PMC7055317; doi:10.1038/s41598-020-60640-z)
Supplement: Supplementary file 1 — Supplementary information. [file 41598_2020_60640_MOESM1_ESM.docx]

**Supplementary Information**

**Iron redox pathway revealed in ferritin via electron transfer analysis**

Peng Chen^1^, Evelien De Meulenaere^2^, Dimitri D. Deheyn^2,*^, Prabhakar R. Bandaru^1, *^

^1^Department of Mechanical Engineering, University of California, San Diego,

La Jolla, CA, 92093, USA

^2^Marine Biology Research Division, Scripps Institution of Oceanography,

University of California, San Diego, La Jolla, CA 92037, USA.

**Table S1.** Concentration (in ng/mL, or ppb) of total iron (^57^Fe) measured from worm and human ferritin samples using ICP-MS. RSD (relative standard deviation) and cps (counts per second) are numbers generated by the instrument during analysis.

| **Sample** | **Concentration average (ppb)** | **Intensity**  **Average (cps)** | **Intensity**  **RSD** | **Concentration RSD** |
| --- | --- | --- | --- | --- |
| **ChF WT** | 15.728 | 110,612 | 1.70% | 3.10% |
| **HuHF WT** | 13.318 | 101,621 | 1.20% | 2.40% |

**Table S2.** Protein concentration range as determined by Bradford method in the ferritin samples and calculation of the amount of iron per protein cage. The range of Fe per ferritin cage is only based on the range in protein concentration as determined by Bradford, not on experimentally determined differences in iron concentration per cage.

| **Sample** | **Protein concentration range as measured by Bradford (mg/mL)** | **Molecular Weight protein monomer (kDa)** | **Protein concentration in 1/100 dilution (monomer) (µM)** | **Protein concentration (cages) (µM)** | **Fe conc. (µM)** | **Fe per protein cage** |
| --- | --- | --- | --- | --- | --- | --- |
| ChF WT | 1.25-1.55 | 19.9 | 0.628-0.779 | 0.0262-0.0325 | 0.276 | 8.50-10.5 |
| HuHF WT | 1.17-1.29 | 21.2 | 0.552-0.608 | 0.0230-0.0254 | 0.234 | 9.21-10.2 |

**Section 1: Control experiments confirming that the observed redox signals were specific to Fe ions in ferritin.**

The DPV of HuHF, ChF and commercialized available Equine spleen Apoferritin (ApF) are indicated in **Figure S1,** below. The cathodic and anodic peak amplitudes of HuHF and ChF are much higher than Apoferritin, corresponding to the greater abundance of iron, in the former. The correspondence to specific A and B sites, relevant to HuHF and ChF, respectively are inferred from the relative position of the anodic peak/s (as indicated in the main text). The negligible ApF related signal indicates the absence of iron. It may be concluded then that the related detected signals for ChF and HuHF (in Figures 2-4 of the main text) are ferritin specific.

**
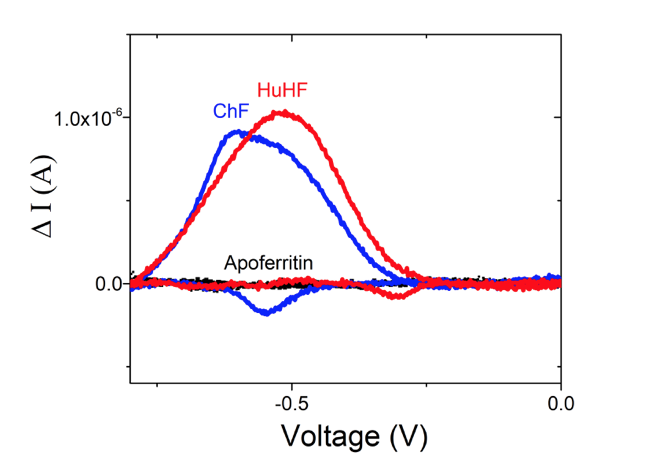
**

**Figure S1.** Comparison of the DPV spectra of HuHF (**red**), ChF (**blue**) and Apoferritin (**black**)

**Section 2: Site specificity of the A and B sites in HuHF and ChF**

The specificity of A and B sites, relevant to HuHF and ChF, in the electrochemical analyses, were inferred from the relative position of the anodic peak/s, where A (/B) is more electro-positive (-/negative) due to the N-(-/O) coordination.


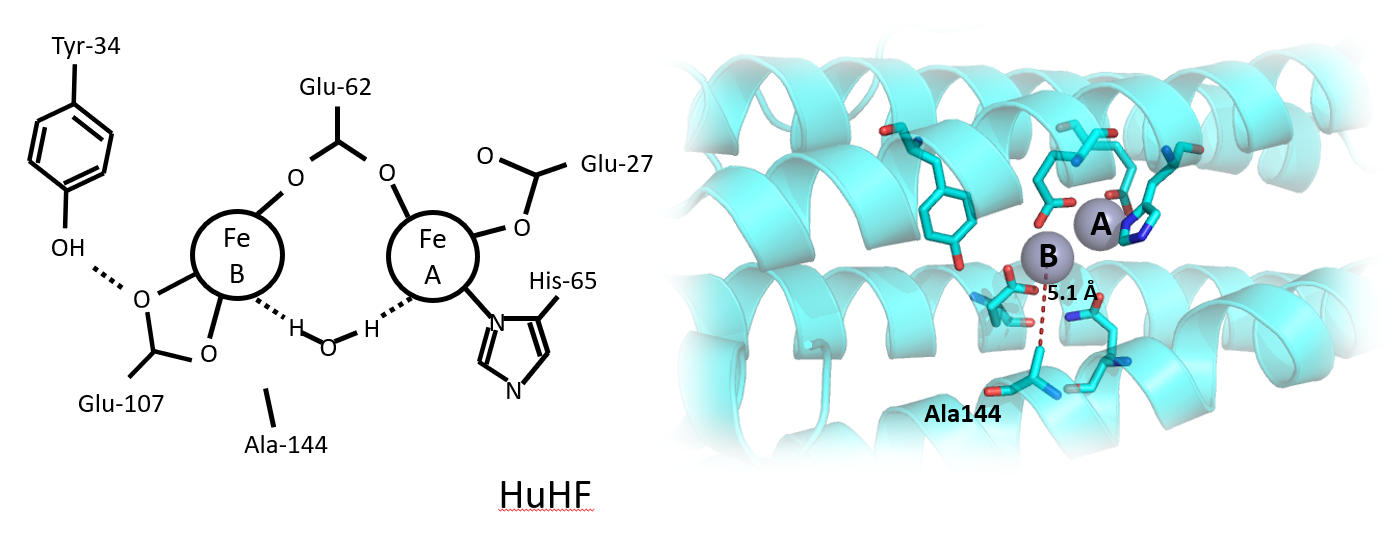


**Figure S2.** The coordination environment of the A and B sites in HuHF – also see Figures 1(c) and 3(a), in the main text.


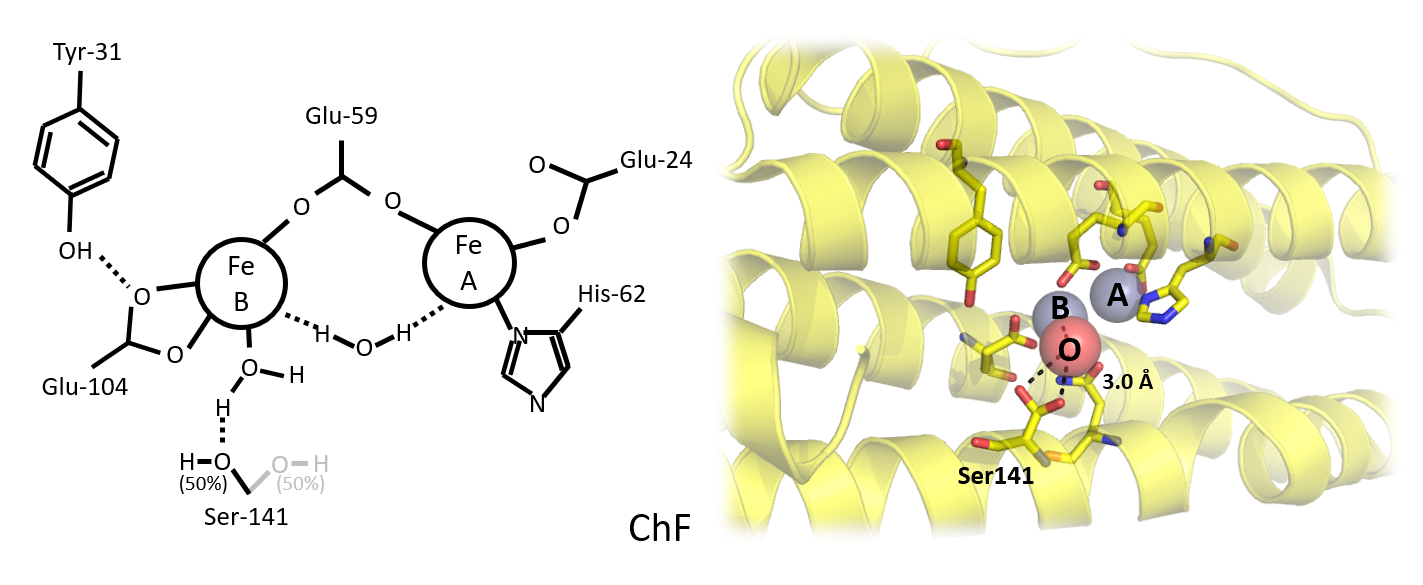


**Figure S3.** The coordination environment of the A and B sites in ChF – also see Figures 1(c) and 4 (a), in the main text. 5WPN shows ~50% dual conformation for Ser-141 (indicated in grey).


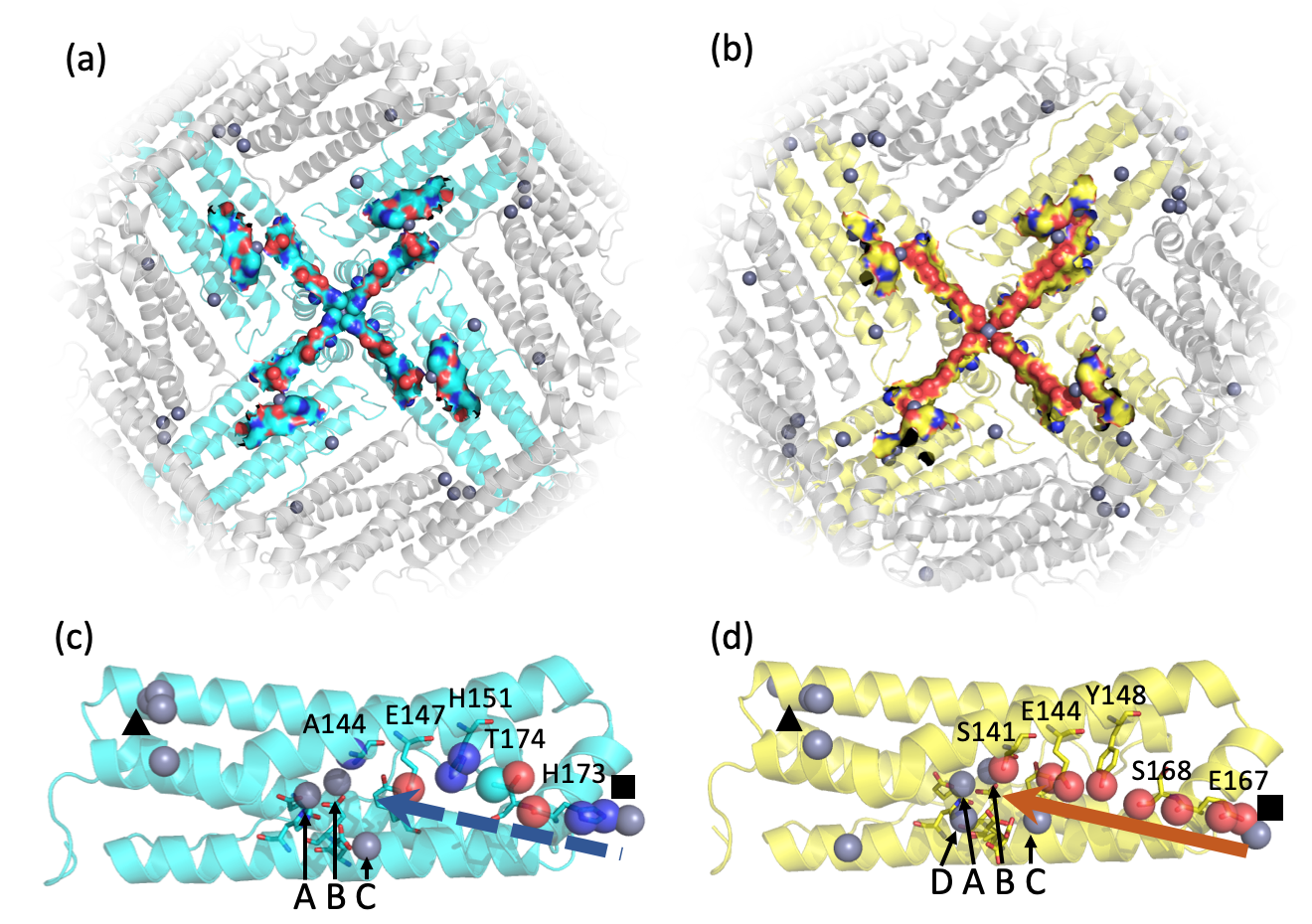


**Figure S4. (a)** HuHF cage viewed from the inside of the sphere up the four-fold axis, with four subunits in cyan and the surface of the residues in the path between the four-fold channel and the respective ferroxidase sites. The residues are displayed through “sticks” and their available atoms at the inside surface of the ferritin shell are displayed in spheres*, i.e.,* Red: oxygen, dark blue: nitrogen, cyan: carbons in HuHF, grey spheres: metal ions. **(b)** ChF cage viewed down the four-fold axis, with carbons in yellow. A clear bright red path is extant between the four-fold channel and the ferroxidase sites. **(c)** and **(d)** are monomers of HuHF and ChF, respectively. The red spheres in **(d)** show the hydroxide groups available for binding Fe (grey spheres) on its way to the ferroxidase center. In (c) corresponding atoms are represented in spheres (color coding – see above), only a minority of which are OH groups. The arrows indicate the direction of putative ion flow towards the ferroxidase center. A black square (■) and triangle (▲) are added to indicate where the four- and three-fold symmetry axes or channels are respectively located, within the subunit. All figures were generated in PyMol^1^. 2CIH was used for HuHF and 5WPN for ChF.

**Section 3. Site specificity of the D sites**

Integral to the alternative pathway is another metal binding site - arbitrarily labeled “D” in Figure 4(a), proximate to the A site, and found in the ChF crystal structure^2^. Comparison with over 30 other available crystal structure of eukaryotic (Heavy and Middle chain) ferritins shows that this is a unique configuration (List of pdbs compared include 1EUM, 1FHA, 2FHA, 2CLU, 2CEI, 2CIH, 2CHI, 2CN7, 2CN6, 2IU2, 2Z6M, 3AJO, 3AJQ, 3AJP, 3KA4, 3A68, 3ERZ, 3RE7, 4DZO, 4DYZ, 4DYY, 4DYZ, 4OYN, 4Y08, 4YKH, 4ZJK, 4ISM, 5CMQ, 5CMR 5UP7, 5UP8, 5UP9, 5VTD). As is often done in enzyme crystallography, Zn^2+^ was used as an inert substrate alternative with comparable size and charge comparable to Fe^2+^ in the generation of the 5WPN crystal structure^2,3^. While this has proven to be a good approach, there is a possibility of overinterpretation.


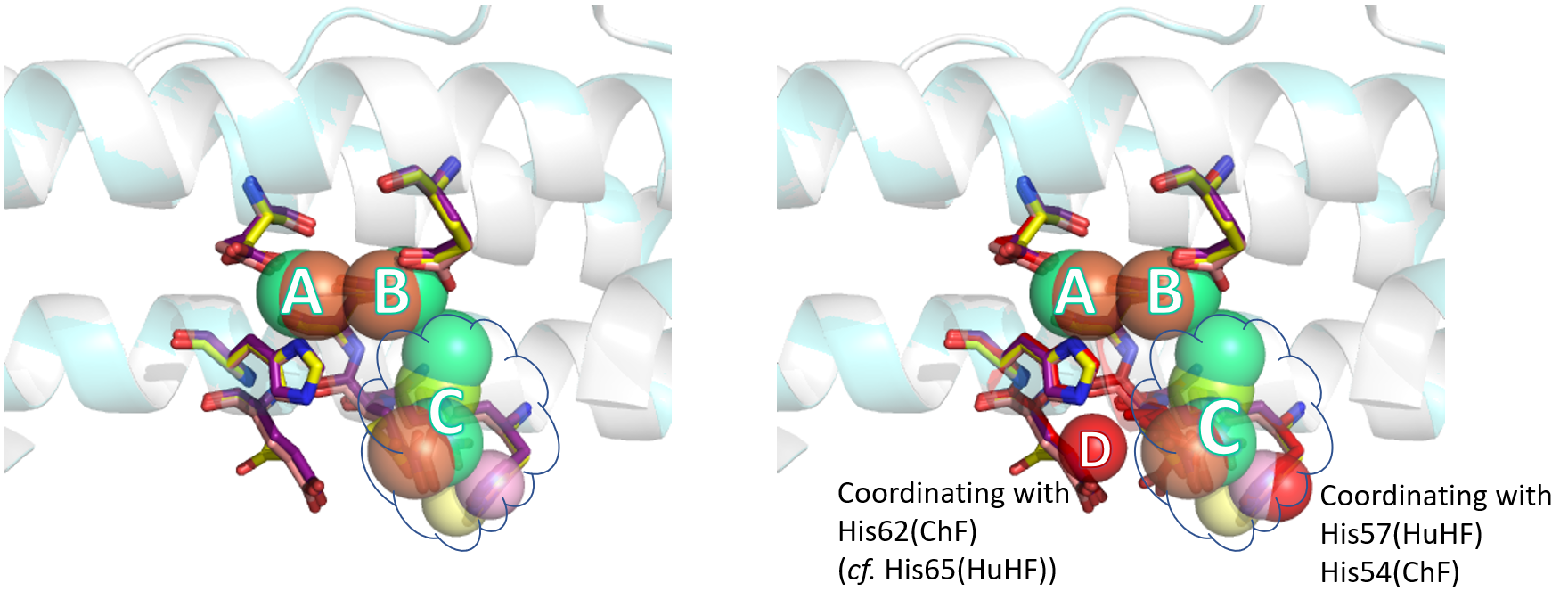


**Figure S5**. Crystal structures of ferroxidase center with A, B and C sites on the left and A – D sites on the right. Backbone and ferroxidase site showing from 2CIH, 3AJO and 5WPN; metal ions from 1FHA, 2CIH, 3AJO, 3KA4, 3RGD, 3A68, 1RO3, 2DJ7. Also conforming with this C site are 1SQ3, 2DJ8, 4ISM and 5CMQ. Spheres in yellow and red are Zn ions from the 2CIH, 3AJO and 5WPN structures, respectively. Green, pink and brown spheres are Ca, Mg, Co (all green), Zn (pink) and Fe (Brown) ions from other structures.

**Section 4. A comparison of the DPV voltammograms of *native* ferritin (with ~ 9-10 Fe per cage) and lightly loaded ferritin (~ 31-32 Fe/cage)**

The DPV scans for native HuHF and native ChF are indicated in **Figure S6** **(a)** and **S6(b),** respectively. Using additional FeCl_2_, it was estimated that the Fe loading was 31-32 Fe per cage. the effects of which on the HuHF and ChF are indicated in **Figures S6(c) and Fig. S6(d),** respectively. The DPV scans look similar to what was observed in the native ferritin case, with enhanced loading


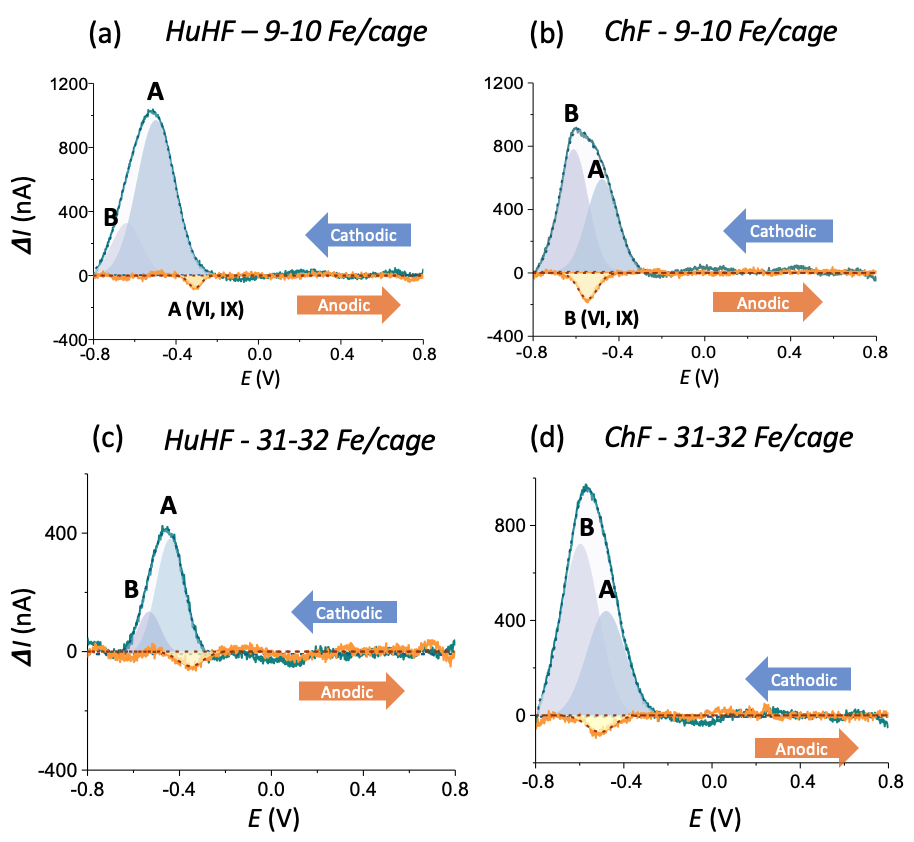


**Figure S6**. (a) – (b) DPV derived peaks for native ferritins (~9 – 10 Fe per cage), for HuHF and ChF, respectively. (c) – (d) DPV for ferritins (with ~ 31—32 Fe per cage) after *enhanced* iron loading using FeCl_2_.

**Reference:**

(1) Schrodinger, L. The PyMOL Molecular Graphics System, Version 1.3 R1. PyMol. **2016**.

(2) De Meulenaere, E.; Bailey, J. B.; Tezcan, F. A.; Deheyn, D. D. First Biochemical and Crystallographic Characterization of a Fast-Performing Ferritin from a Marine Invertebrate. *Biochem. J.* **2017**, BCJ20170681.

(3) Toussaint, L.; Bertrand, L.; Hue, L.; Crichton, R. R.; Declercq, J. P. High-Resolution X-Ray Structures of Human Apoferritin H-Chain Mutants Correlated with Their Activity and Metal-Binding Sites. *J. Mol. Biol.* **2007**, *365* (2), 440–452.
